# A study on the influence of community spiritual comfort service on the mental health of older people

**DOI:** 10.3389/fpubh.2023.1137623

**Published:** 2023-03-10

**Authors:** Jing Dai, Yang Liu, Xue Zhang, Zongyue Wang, Yunjuan Yang

**Affiliations:** ^1^Faculty of Management and Economics, Kunming University of Science and Technology, Kunming, China; ^2^Yunnan Provincial Center for Disease Control and Prevention, Kunming, China

**Keywords:** mental health, spiritual consolation, the mediation effect, health care facilities, emotional characteristics

## Abstract

**Background:**

China is experiencing rapid population aging, with the proportion of older adult people aged 60 and above reaching 19. 8% of the total population in 2022. With the growth of age, the physical function of older adults declines and their mental health is getting worse, with the increasing trend of empty nesting and childlessness, older adults lack information and social interaction with others and fall into social isolation, loneliness and some mental health problems, the proportion of older adults with mental health problems gradually rises and the mortality rate increases incrementally, requiring effective ways to intervene in the mental health of older adults and promote healthy aging.

**Aim of the study:**

The present study investigated the influence of spiritual comfort older adult services on the mental health of 12,624 older adults aged 60 years or older in 23 provinces in China from 2017 to 2018, with the aim of providing a case for the development of more targeted mental health strategies for older people.

**Methods:**

Using the data from the 2018 CLHLS Survey, the influencing factors of the mental health of older people were analyzed using chi-square test and the logit regression model. The mechanism of the health care facilities and the spiritual comfort services on mental health was analyzed using the chain mediation effect.

**Results:**

The spiritual comfort services decreased the risk of negative emotion and mental health of older adults, with female (OR = 1.168), rural residents (OR = 1.385), no drinking (OR = 1.255), not exercising (OR = 1.543), not having pension insurance (OR = 1.233), and low annual household income (OR = 1.416) being the characteristics as risk factors. The mediating effect results showed that the health care facilities had a partial mediating effect between the spiritual comfort services and the mental health status of older people, and the mediating effect accounted for 40.16% of the total effect.

**Conclusions:**

The use of spiritual comfort services can effectively reduce and alleviate the adverse mental health symptoms of older people, promote guidance and health education for healthy older people and chronically ill patients, and improve the good health perception of older people in order to improve their quality of life and mental health status.

## Introduction

In recent years, the aging population has seriously hindered the development of the world economy. China is the largest developing country in the world and have the largest number of older people in the world right now, and this will continue 2,050 and beyond (1). According to statistics, more than 20% of adults aged 60 and over suffer from mental or neurological disorders (2), and the lifetime prevalence rate of any mental health problem in China is 16.6 % (3). The physical and mental health and quality of life of older adults has become an important issue for society, and researchers are increasingly concerned about how care services can support an aging society and promote the lives and health of older adults (4, 5). While older adults fall into social isolation, loneliness and some mental health problems due to retirement, physical health problems and other reasons, older adults lack information and social interaction with others (6, 7). In particular, the blockade and social distance during the COVID-19 led to depression in many older adults (8), who are at higher risk of developing serious diseases, with a mortality rate of 3.6% for those aged 60–69 years, increasing to 18% for those over 80 years (9). In addition, the social consequences of isolation are considered. Social disconnection is particularly important for older adults who are less accustomed to digital technology, as it may limit social activities, interfere with daily life, increase substance use, and reduce sensory stimulation. All these conditions combined with isolation may have a negative impact on the mental health of older adults.

In this context, China's social senior service system proposes to build a social senior service system based on home-based senior care, community-based senior care, institutional senior care as a supplement, and the combination of medical care, and proposes a “9,073” senior service pattern, that is, home care services provided by the family accounts for 90%, senior service provided by the community accounts for 7%, and senior services provided by the institution accounts for 3%.Community senior services, as an important part of senior service policy system, basing on living environment and family, can provide specialized services such as day care, medical rehabilitation, nursing care, spiritual comfort and recreational activities for older people and reduce their physical health burden, especially, spiritual comfort services can promote the social participation of older people, as well as improve their sense of health and wellbeing and access. The spiritual comfort demand of older people refers to the psychological needs of older people in terms of emotional communication, social interaction and self-actualization after retirement in order to relieve their misery, change their monotonous life and realize their own value (10). Some scholars have explored the role of community spiritual comfort senior services on the quality of life of older people, and concluded that community spiritual comfort senior services can have a positive impact on the wellbeing of older people and significantly improve their mental health (11). Compared to older adults who do not enjoy community services, those who receive community-based spiritual comfort services have higher life satisfaction (12), and a well-established supportive community environment for older adults can effectively alleviate the adverse effects on mental health and has a significant psychological building effect (13), thus reducing their negative psychological problems such as loneliness and helplessness, depression, anxiety and depression, which have a positive impact on improving the quality of life of older people and extending their healthy life span.

Mental health has always been a prominent problem for older people in China, and scholars have begun to pay attention to the factors influencing the mental health of older people, in an effort to gain a more comprehensive understanding of the root causes of their mental problems, address their mental illnesses, and improve their mental health. The factors affecting the mental health of older people can be summarized as individual characteristics, lifestyle habits (smoking, drinking, exercise, etc.), social relationships (family relationships, interpersonal relationships, etc.), economic status (income level, social security), etc. The mental health status of older adults tends to vary depending on the type of residence or the way they age. Compared to institutional care, the mental health of older adults who age at home and in the community is significantly better (14), and living with their daughters is the most beneficial to the mental health of older adults (15). In terms of social factors, some scholars' studies found that social support from family, neighbors, and friends influenced older adults' subjective wellbeing and mental health by affecting their sense of self-esteem and loneliness (16). In terms of economic factors, Su Hong's study all showed that economic income had a positive impact on the psychological wellbeing of empty- and non-empty-nesting older adults (17). The coping capacity of older adults in this study includes health care facilities at both the family and social levels, health care facilities at the family level reflects the informal care of the older adults by relatives and friends, and health care facilities at the social level reflects the function of mutual help and risk regulation (e.g., social support, medical accessibility), and the improvement of the health care facilities of older adults is important for alleviating or regulating the mental health condition of older adults (18).

This study explores the factors influencing the mental health of older adults and the mechanism of the influence of spiritual comfort services on the mental health of older adults using data from the Chinese Longitudinal Healthy Longevity Survey (CLHLS) 2018. The aim is to alleviate the mental health vulnerability of older people, improve their mental health and quality of life, and provide some reference for vigorously promoting “healthy aging” and improving the mental health of older people in China.

## Theoretical basis

Research on the relationship between community-based spiritual comfort services and the mental health status of older people has started earlier by domestic and foreign scholars, and related studies mainly hold positive theories. It has been shown that health interventions in spiritual comfort services improve the self-rated health status of older adults, with general improvements in mental health and a reduced incidence of anxiety (19), contributing to improved physical and mental health, wellbeing, and social support for vulnerable populations. A study of senior depressed patients found that community-based spiritual comfort services effectively managed senior patients from medical, functional, and social perspectives, which not only contributed to the early detection and treatment of depression, but also to the rational use of primary care resources (20). In addition, the provision of community-based spiritual comfort services contributes to improving the quality of care and life of older adults and enhancing their physical functional capacity, while increasing communication with the outside world, diminishing the sense of loneliness and isolation, and making a significant contribution to improving their mental health.

Social support can facilitate the exchange of psychosocial resources for older individuals and frail individuals, such as emotional or instrumental support among members of society, thus promoting mental health (21). At the community level, social support enables older age groups to organize and act collectively, and facilitates the creation of new social ties and cohesion. The findings suggest that there is an increasing tendency for older adults to live alone due to changes in demographics, social welfare systems, and family culture, and that older adults' health is not necessarily at risk if they maintain social ties outside the home and avoid social isolation. Loneliness and social isolation may also lead to many problems such as depression, cognitive dysfunction, disability, cardiovascular disease, further exacerbating pre-existing health conditions, and increased mortality in older adults (22).

**Figure 1:**
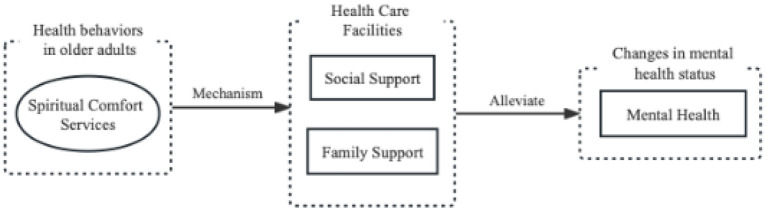
Theoretical framework diagram.

Therefore, health care facilities has become an important influencing factor on the health behavior, emotional health, and cognitive level of older adults from the current study, and may be an important mediator of the level of mental health between community-based spiritual comfort services and older adults, as well as an explanatory mechanism by which the provision of community-based spiritual comfort services affects the mental health of older adults (Figure 1).

## Materials and methods

### Data source

**Figure 2:**
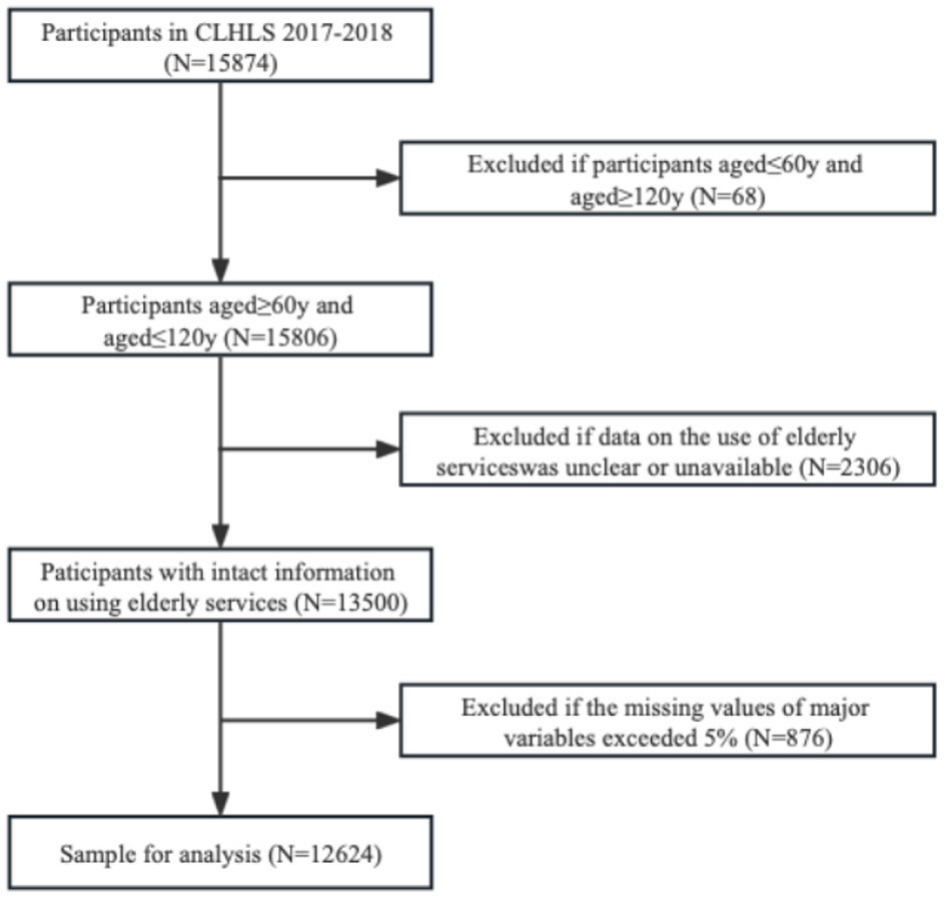
Flowchart of the study sample selection.

The data used in this study were obtained from the Chinese Longitudinal Healthy Longevity Survey (CLHLS), which was organized and implemented by the Center for Healthy Aging and Development Research/National Development Research Institute of Peking University. The survey covered 23 provinces across China, and the survey population was mainly older people aged 60 years and above. The survey mainly collected data related to basic information, socio-economic background and family structure, economic sources and economic status, self-assessment of health and quality of life, cognitive function, personality characteristics, daily activity ability, lifestyle, life care, disease treatment and medical cost bearing of older people and their families. A total of 15,874 older adults aged 60 years and older were surveyed in the CLHLS database in 2017–2018, and information on individual physical health indicators such as daily mobility, subjective feelings, and physical functional status, individual personality and emotional perceived mental health indicators, community senior services, and the health care facilities of older adults were selected as study variables in this study, samples with a large number of missing values for the main variables and samples with outliers over 120 years of age were removed, and data with missing values < 5% of the overall sample were filled by interpolation in Stata 15.0 in order to maximize the use of available survey information, resulting in a valid sample size of 12,624. This study used SPSS25.0 for the internal consistency of the demand for community-based spiritual comfort services, and the result was Cronbach's coefficient of 0.828 (Table 1), indicating that the questionnaire passed the reliability analysis. Validity analysis is to verify the validity of the data, and is used to detect whether the results of the questionnaire measurement can correctly reflect things. SPSS25.0 was used to test the validity of community spiritual comfort services, and the KMO was 0.872, and the significance of Bartlett's sphericity test was *P*=0.000, indicating that the questionnaire has high validity (Table 2). The detailed sample selection process is presented in Figure 2.

**Table 1:** Reliability analyze.

**Cronbach‘s alpha**	**Item**
0.828	3

**Table 2:** Validity test of community spiritual comfort service.

**KMO**		**0.872**
Bartlett test of sphericity	Approximate chi-square	6,880.660
	df	3
	Sig.	0.000

### Measurement of mental health

Based on the existing methods of measuring mental health of older people, the mental health of older people was divided into four dimensions of emotional characteristics, geriatric anxiety, geriatric depression and cognitive impairment to measure the mental health vulnerability representations of older people (18). Among them, the emotional characteristics were measured by the CLHLS questionnaire “Can you always think about whatever happens to you? Depression was assessed by the CESD-10 scale, and a score of < 10 was considered as having no depressive symptoms, and a score of more than or equal to 10 was considered as having depressive symptoms (23), the scale's Cronbach's alpha was an excellent 0.836 in the current sample. Anxiety was assessed by the GAD-7 scale, and a score of < 10 was considered as having no anxiety symptoms, and a score of more than or equal to 10 was considered as having anxiety symptoms (24), the scale's Cronbach's alpha was an excellent 0.927 in the current sample. Cognitive impairment was assessed by using four modules of CLHLS: general ability, reaction ability, attention and calculation ability, recall, language, comprehension and self-coordination ability, and the correct answers were summed up, the scale's Cronbach's alpha was an excellent 0.936 in the current sample (Table 3). Meanwhile, JunNing Fan's index calculation method was referred to calculate the mental health index of older people, in order to examine the characteristics of mental health of older people, and each variable was dichotomized or mapped to the 0.00–1.00 interval, with 0.00 indicating no deficits (the healthiest state) and 1.00 indicating the greatest deficits (the least healthy state), while the index was calculated by summing the deficit scores of the different dimensional indicators of the older individual divided by the highest score of the four indicators included, according to the consensus of constructing the index, without assigning weights to variables related to each other (25), which was used to respond to the mental health representations of older individuals, with a mental health index >0.25 being classified as fragile and a mental health index < 0.25 being considered healthy.

**Table 3:** Indicator definition and assignment.

**Values**		**Indicator definition**	**Variable assignment**
Mental health	Cognitive impairment	Do you always look on the bright side of things?	Always = 0, often = 0.25, sometimes = 0.5, seldom = 0.75, never = 1
	Geriatric depression	CESD-10 scale	Score ≤ 10 points is 0, score>10 is 1
	Geriatric anxiety	GAD-7 scale	Score ≤ 10 points is 0, score>10 is 1
	Emotional Characteristics	Orientation, registration, attention and calculation, recall, language	Add up the correct answers and assign a score of 1 for 0–10, 0.75 for 11–16; 0.5 for 17–20, 0.25 for 21–23, 0 for 24
Health care facilities	Medical accessibility	Can you get adequate medical service when you are sick?	Yes = 1, No = 0
	Routine medical examination	Do you have regular physical examination once every year?	Yes = 1, No = 0
	Pension insurance	Do you participant in public old age insurance?	Yes = 1, No = 0
	Care by family and friends	When you are sick, is there someone to take care of you?	Yes = 1, No = 0
	Living alone or not	Co-residence	Yes = 1, No = 0

### Socio-demographic information

This includes demographic characteristics (age, sex, type of household, marital status), family status (living style, care by relatives and friends), lifestyle (smoke or not, drink or not, whether to exercise), economic status (pension insurance, social and commercial insurance, annual household income). Based on existing studies, five dimensions of the CLHLS questionnaire were used to define the health care facilities of older people: routine medical checkups, pension insurance, accessibility to medical care, living alone, and care by family and friends. Among them, ”Do you have a regular medical checkup once a year¿‘ as a measure of regular medical checkups, pension insurance based on the question ”Are you enrolled in pension insurance¿‘ and ”If you are seriously ill, can you get to the hospital in time¿‘ as a measure of medical accessibility; whether you live alone based on the question ”Do you currently live alone¿‘ measured by the question ”Currently, do you have someone to take care of you when you are not feeling well or when you are sick¿‘ measures care by a friend or relative and is assigned a value of 1 if the older person has this coverage, otherwise it is assigned a value of 0.

Spiritual comfort senior service includes spiritual comfort chatting and relief service, organizing social and recreational activities service, and dealing with family and neighborhood disputes service. The independent variable was whether older people had used spiritual comfort services, if older people have used at least one of three seniors care services, the value is 1, otherwise the value is 0.

### Statistical methods

Stata 15.0 was used for data collation and statistical analysis, descriptive statistical analysis and logit regression analysis were conducted on the individual characteristics, family status, lifestyle, and economic situation of the study subjects, chi-square tests were used to examine the general characteristics differences between healthy and unhealthy subjects according to the distribution of categorized variables. Count and percentages were used to describe categorical variables, and Amos 23.0 was used to establish a structural equation model to analyze the influence mechanism of mental health of older people, and to explore the influence of spiritual comfort type of senior services on the mental health status of older people. The difference was considered statistically significant at *P* < 0.05.

### Model construction

This study used logit regression modeling and structural equation modeling, respectively, in order to investigate the mechanism of the effect of spiritual comfort type of senior services on the mental health of older people.

Among the explanatory variables of the logit model were emotional characteristics of older adults, geriatric depression, geriatric anxiety, and cognitive impairment. The model expressions are as follows.


(1) $$\begin{array}{l} Health_{i}=\beta_{0}+\beta_{1}Spiritual_{i}+\sum_{j=2}\beta_{j}X_{i}+\varepsilon \end{array}$$

where *Health*_*i*_ represents the emotional characteristics, geriatric depression, geriatric anxiety and cognitive impairment of older people, β_0_ is an intercept term, *Spiritual*_*i*_ represents spiritual comfort type of senior services, *X*_*i*_ represents sociodemographic variables including Individual Characteristics, Family Status, Lifestyle and Economic Status, and ε is a random disturbance term (26).

The structural equation model is based on a regression model to statistically analyze the latent variables and to verify whether the relationship between the latent variables is consistent with the reality. The structural equation consists of a measurement model and a structural model. Equations (2) to (4) are the measurement model and equation (5) is the structural model, and the relationships are as follows (27).


(2) $$\begin{array}{l} Spiritual_{i}=\Lambda_{Spiritual_{i}}X_{1}+\delta_{1} \end{array}$$

(3) $$\begin{array}{l} Ability_{i}=\Lambda_{Ability_{i}}X_{2}+\delta_{2} \end{array}$$

(4) $$\begin{array}{l} Health_{i}=\Lambda_{Health_{i}}X_{3}+\varepsilon \end{array}$$

*Spiritual*_*i*_ represents access to the ith type of spiritual comfort type of senior service, *Ability*_*i*_ represents access to the ith type of health care facilities, and *Health*_*i*_ represents the ith type of mental health of older people.*Spiritual*_*i*_ and *Ability*_*i*_ are indicators of exogenous latent variables, *Health*_*i*_ is an indicator of endogenous latent variables, Λ_*Spiritua*_*l*__*i*__, Λ_*Abilit*_*y*__*i*__ denote the relationship between *Spiritual*_*i*_, *Ability*_*i*_ and *X*_*i*_, respectively, Λ_*Healt*_*h*__*i*__ denotes the relationship between *Health*_*i*_and *Y*, and δ_*i*_ and ε are the errors on the measurement of latent variable indicators.


(5) $$\begin{array}{l} Y={\text{Γ}}_{i}X_{i}+\varsigma \end{array}$$

where Y denotes the endogenous latent variable, *X*_*i*_ denotes the exogenous latent variable, Γ_*i*_ denotes the effect of the ith exogenous latent variable on the endogenous latent variable, and ς denotes the part of the model that fails to be explained within the model.

## Results

### Mental health status of different older adults

The proportion of older women with poorer mental health is greater than that of older men, the older an older person is, the more fragile their mental health status becomes; in terms of household type, older adults in urban households have more optimistic mental health status than those in rural households. The mental health of older people who are divorced, widowed or unmarried is worse than that of those who have a partner. Older adults without pension insurance were more likely to be mentally unhealthy, and those with an annual household income of more than 60,000 yuan had better mental health than those with an annual household income of < 10,000 yuan and those with an annual household income of 10,000 yuan to 60,000 yuan (Table 4). It is clear from this that as older adults age and lose their roles, widowhood, retirement, etc., older adults reduce their activity level and their contact with others, leading to a loss of social connections that can lead to psychological problems such as loneliness, anxiety, and depression.

**Table 4:** Basic mental health status of different older adults.

**Variables**		**Mental health status (%)**		**χ^2^**	***P*-value**
		**Health**	**Vulnerability**		
Individual characteristics	Age			255.47	< 0.001
	60~69 year old	1,131 (73.78%)	402(26.22%)		
	70~79 year old	2,542 (71.09%)	1,034 (28.91%)		
	≥80 year old	4,357 (57.98%)	3,158 (42.02%)		
	Sex			189.00	< 0.001
	Male	4,153 (70.80%)	1,713 (29.20%)		
	Female	3,877 (57.37%)	2,881 (42.63%)		
	Household type			79.58	< 0.001
	Urban	2,545 (69.80%)	1,101 (30.20%)		
	Rural	5,485 (61.09%)	3,493 (38.91%)		
	Marital status			282.44	< 0.001
	Married	4,229 (71.39%)	1,695 (28.61%)		
	Single	3,801 (56.73%)	2,899 (43.27%)		
Family status	Residence type			67.58	< 0.001
	Living with family	6,612 (65.24%)	3,523 (34.76%)		
	Living alone	1,203 (57.64%)	884 (42.36%)		
	Senior care facilities	215(53.48%)	187 (46.52%)		
	Care by family and friends			17.59	< 0.001
	Yes	8,394 (67.83%)	3,981 (32.17%)		
	No	137(55.24%)	111(44.76%)		
Lifestyle	Smoking or not			47.72	< 0.001
	Yes	1,488 (71.37%)	597(28.63%)		
	No	6,542 (62.07%)	3,997 (37.93%)		
	Drinking or not			90.17	< 0.001
	Yes	1,486 (74.30%)	514 (25.70%)		
	No	6,544 (61.60%)	4,080 (38.40%)		
	Whether to exercise			442.30	< 0.001
	Yes	3,312 (75.56%)	1,071 (24.44%)		
	No	4,718 (57.25%)	3,523 (42.75%)		
Economic status	Pension insurance			73.08	< 0.001
	Yes	3,347 (67.49%)	1,612 (32.51%)		
	No	4,683 (61.10%)	2,982 (38.90%)		
	Social security and commercial			57.6	< 0.001
	Yes	198 (65.56%)	104 (34.44%)		
	No	8,333 (67.63%)	3,988 (32.37%)		
	Annual household income			249.77	< 0.001
	Under 10,000 RMB	1,933 (59.62%)	1,309 (40.38%)		
	10,000 RMB-60,000 RMB	3,419 (68.48%)	1,574 (31.52%)		
	Over 60,000 RMB	3,179 (72.45%)	1,209 (27.55%)		

### Regression results analysis of factors influencing mental health of older people

In this study, the independent variables that passed the chi-square test were placed in the regression model as the dependent variable of correlating, and the regression results are shown in Table 5. Emotional characteristics in the mental health of older people are significantly affected by the spiritual comfort services, however, the effects on geriatric depression, geriatric anxiety and cognitive impairment were not significant. Older adults who had access to spiritual comfort type of senior services had 90.5% of the risk of having negative emotional characteristics as those who did not have access. For the emotional characteristics of older adults, sex, type of household registration, care by family and friends, whether they drink alcohol, whether they exercise, pension insurance, social and commercial insurance, and annual household income had significant effects on emotional characteristics. The risk of negative mood among female older adults was 1.168 times higher than that of male older adults, the risk of negative mood among rural older adults was 1.385 times higher than that of urban older adults, the risk of negative mood among older adults who drank alcohol regularly and exercised regularly was 0.797 and 0.648 times higher than that of older adults who did not drink alcohol or exercise respectively, the risk of negative mood among older adults with pension insurance and social insurance was 0.811 and 0.648 times higher than that of older adults without insurance, respectively. The higher the annual household income, the lower the risk of negative emotion among older people.For depressive symptoms in older adults, age, sex, type of household registration, marital status, residence type, care by family and friends, whether or not they drink alcohol, whether or not they exercise, pension insurance, social and commercial insurance, and annual household income had a significant effect on depression in older adults. The risk of depression was 1.304 times higher among female seniors than male seniors, 1.198 and 1.504 times higher among older people living alone or in a nursing facility than among older people living with family members, respectively, 0.487 times higher among older people being cared for by friends and relatives than among older people not being cared for by friends and relatives. The risk of depression among older people who drink alcohol and exercise regularly is 0.759 and 0.519 times higher than that of older people who do not drink alcohol or exercise, respectively, and the risk of depression among older people who have pension insurance is 0.841 times higher than that of older people who do not have insurance. The higher the annual household income, the lower the risk of depression in older adults. For anxiety symptoms in older adults, sex, care by family and friends, whether or not they smoked, whether or not they exercised, and annual household income had significant effects on anxiety in older adults. The risk of anxiety in female seniors was 1.706 times higher than that in male seniors, the risk of anxiety among older people who were cared for by family and friends was 0.172 times higher than that among older people who were not cared for by family and friends, the risk of anxiety among older people who smoked regularly and exercised regularly was 0.511 and 0.549 times higher than that among older people who did not smoke or exercise, respectively, and the higher the annual household income, the lower the risk of anxiety in older people. For cognitive impairment in older adults, age, sex, household type, marital status, residence type, whether or not they drink alcohol, whether or not they exercise, pension insurance, and annual household income had significant effects on cognitive impairment. The risk of cognitive impairment was higher for older adults, 1.708 times higher for female than male adults, lower for single than married adults, lower for those living alone, and higher for those living in a nursing facility, the risk of cognitive impairment was 0.656 times higher in older adults who exercised regularly than in those who did not, and the risk of cognitive impairment was greater in those with pension insurance. The higher the annual household income, the lower the risk of cognitive impairment in older adults.

**Table 5:** Regression results of mental health level of older people.

**Variables**		**Emotional characteristics**	**Geriatric depression**	**Geriatric anxiety**	**Cognitive impairment**
Spiritual comfort services		0.905^***^	0.993	1.026	1.049
Individual characteristics	Age (60~69 year old)				
	70~79 year old	0.979	1.078	0.863	1.641^***^
	≥80 year old	0.896	1.232^**^	0.875	6.133^***^
	Sex (male)				
	Female	1.168^***^	1.304^***^	1.706^***^	1.708^***^
	Household type (urban)				
	Rural	1.385^***^	1.109^*^	1.212	1.444^**^
	Marital status (married)				
	Single	1.071	0.860^**^	0.892	0.511^***^
Family status	Residence type (living with family)				
	Living alone	1.031	1.198***	0.910	0.599^***^
	Senior care facilities	1.226^*^	1.504^***^	0.909	1.294^**^
	Care by family and friends (No)				
	Yes	0.742^*^	0.487^***^	0.172^***^	1.129
Lifestyle	Smoking or not (No)				
	Yes	0.936	0.953	0.511^**^	0.995
	Drinking or not (No)				
	Yes	0.797^***^	0.759^***^	0.949	0.871^*^
	Whether to exercise (No)				
	Yes	0.648^***^	0.519^***^	0.549^***^	0.656^***^
Economic status	Pension insurance (No)				
	Yes	0.811^***^	0.841^***^	1.091	1.171^***^
	Social security and commercial (no)				
	Yes	0.686^***^	0.871	0.979	1.137
	Annual household income (under 10,000 RMB)				
	10,000 RMB-60,000 RMB	0.818^***^	0.707^***^	0.647^**^	0.797^***^
	Over 60000RMB	0.706^***^	0.698^***^	0.596^**^	0.695^***^
*N*		12,624	12,624	12,624	12,624

^*^P < 0.05, ^**^P < 0.01, ^***^P < 0.001. The values in the table are OR values.

### Mechanism of the role of spiritual comfort services and health care facilities in influencing mental health of older people

**Figure 3:**
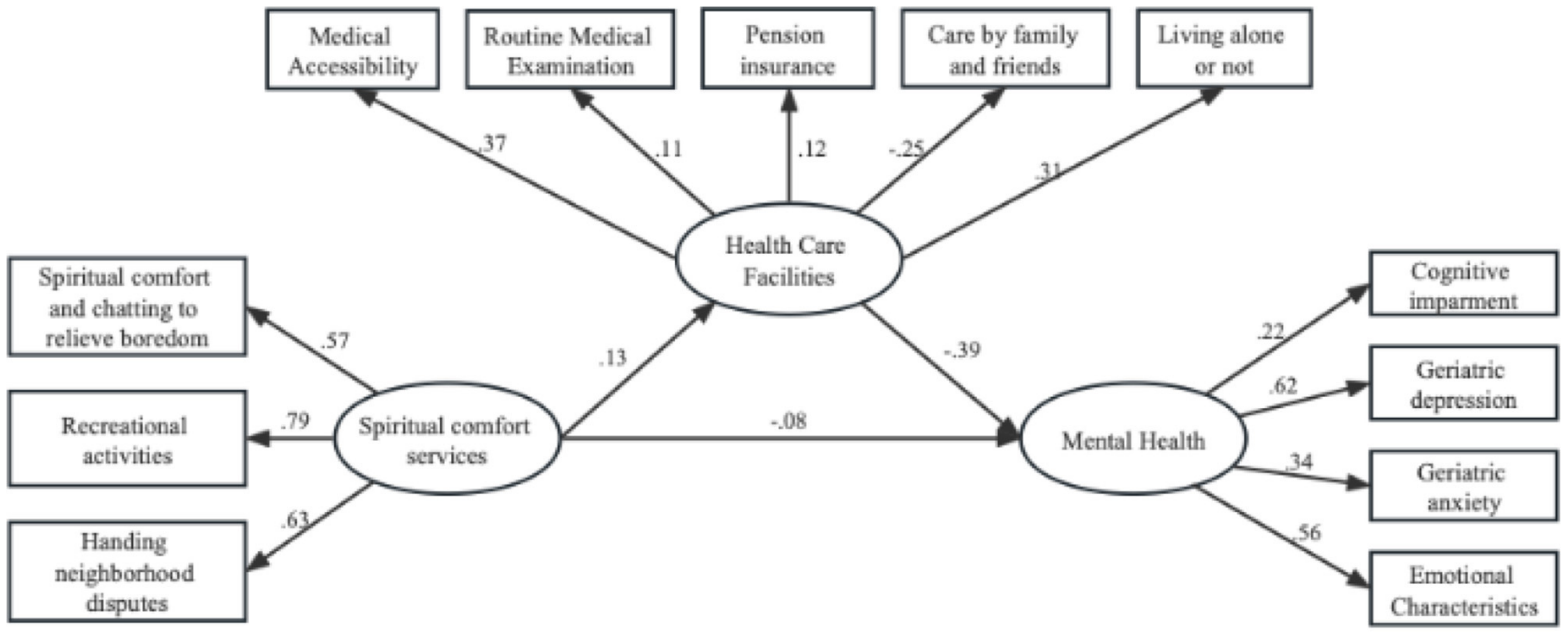
Mechanisms of mental health impact pathways in older adults.

The model fit test was first conducted, and the results showed that the model fit was ideal with χ^2^/df = 19.079, GFI = 0.987, AGF = 0.980, and RMSEA = 0.038. Figure 3 reports the standardized coefficients of the structural equation model, and the results show that the standardized regression coefficients of the spiritual comfort services on the health care facilities of older people are significant and positive, and the standardized regression coefficients of the health care faclities on the mental health are negative. This shows that spiritual comfort services can alleviate negative psychological emotions such as anxiety and depression of older people by influencing their health care facilities.

According to the mental health mechanism and research framework of older people, the relationship between the three latent variables was verified in this study, and the parameters were estimated by the maximum likelihood method, and the structural model estimation results are shown in Table 6. Specifically, the regression coefficient of spiritual comfort services on health care facilities was significantly positive, implying that spiritual comfort services may enhance the health care facilities of older people, which in turn may have some impact on their mental health; meanwhile, the regression coefficient of health care facilities on the mental health of older people is significantly negative, indicating that the mental health condition of older people who lack health care facilities will be more serious, and mental health status of older adults deteriorates as health care becomes weaker. The regression coefficient of the spiritual comfort services on the psychological health of older people was significantly negative, indicating that the spiritual comfort services directly affected the psychological health of older people. There were significant correlations between the three latent variables and the corresponding observed variables, and each 1-unit increase in the service for dealing with neighborhood disputes, the service for recreational activities, and the service for spiritual comfort and chatting to relieve boredom increased the mental health of the older adults by 0.630, 0.791, and 0.572, respectively, as shown by the results of the measurement model estimation (Table 7). Community spiritual comfort services help older people to give them social support in their familiar environment and without severing their original social network, so that they can more easily gain a sense of belonging and security, alleviate the negative psychology brought about by a sense of uselessness and frustration, thus maintaining a positive psychological mood and a healthy mindset in life, and improve their physical and mental health. It is noteworthy that the significant coefficient of the observed variable whether the potential variable health care facilities corresponds to living alone is negative, revealing that older people living alone do not live with their own relatives and children, lack communication with their children, and may experience loneliness, depression, spiritual emptiness, lack of solace, and also more likely to lack a sense of security and belonging.

**Table 6:** Structural equation model estimation results.

**Paths**	**Estimate**	***SE***	***CR***	***P*-value**
Spiritual comfort services → Mental health	−0.076	0.020	−4.772	*P* < 0.001
Health care facilities → Mental health	−0.394	0.050	−9.632	*P* < 0.001
Mental comfort services → Health Care Facilities	0.128	0.028	6.112	*P* < 0.001

The standard coefficients are reported in the table.

**Table 7:** Measurement model estimation results.

**Potential variables**	**Observed variables**	**Estimate**	***SE***	***CR***	***P*-value**
Spiritual comfort services	Handing neighborhood disputes	0.630	0.009	-	*P* < 0.001
	Recreational activities	0.791	0.010	44.561	*P* < 0.001
	Spiritual comfort and chatting to relieve boredom	0.572	0.011	47.059	*P* < 0.001
Health care facilities	Medical accessibility	0.369	0.035	-	*P* < 0.001
	Routine medical examination	0.106	0.022	6.159	*P* < 0.001
	Pension insurance	0.116	0.024	6.662	*P* < 0.001
	Care by family and friends	0.307	0.048	11.546	*P* < 0.001
	Living alone or not	−0.249	0.032	−10.894	*P* < 0.001
Mental health	Cognitive impairment	0.224	0.013	-	*P* < 0.001
	Geriatric depression	0.619	0.015	17.063	*P* < 0.001
	Geriatric anxiety	0.339	0.017	15.910	*P* < 0.001
	Emotional characteristics	0.555	0.015	17.286	*P* < 0.001

The standard coefficients are reported in the table.

### Analysis of the role of intermediaries

This study explored the mediating effect between spiritual comfort services and the mental health of older people through health care facilities, using Bootstrap confidence interval estimation method for interval estimation, selecting 95% confidence interval and repeating 5,000 times to test the mediating effect between spiritual comfort services and the mental health of older people, the direct, indirect and total effects of spiritual comfort type of senior services on the mental health of older people are shown in Table 8.

**Table 8:** Bootstrap model test results.

**Effect**	**Paths**	**Estimate**	**Bias-corrected percentile Bootstrap**	
			**95%*****CI*** **LI**	**95%*****CI*** **UI**
Total effect	Spiritual comfort services → Mental health	−0.127^***^	−0.152	−0.100
Direct effect	Spiritual comfort services → Mental health	−0.076^**^	−0.111	−0.032
Indirect effect	Spiritual comfort services → Mental health	−0.051^***^	−0.094	−0.028

^*^P < 0.05, ^**^P < 0.01, ^***^P < 0.001.

As can be seen in Table 8, the total effect in the path from spiritual comfort services to mental health is −0.127, the direct effect is −0.076, and the indirect effect is −0.051, and the results are all significant at the 0.05 level of significance, indicating that there is a significant effect of the provision of spiritual comfort services on the mental health of older people, and there is a partial mediating effect; indicating that spiritual comfort services indirectly affects mental health by influencing the health care facilities of older people; it confirms that the spiritual comfort services mainly has an effect through the health care facilities mediating variable, and the health care facilities significantly improves the mental health of older people and has a mitigating and inhibiting effect on their mental health vulnerability.

## Discussion

This study analyzed the mental health status of older adults and related influencing factors in China based on the CLHLS 2018 national survey data, which showed that the individual characteristics, family status, lifestyle and economic status of older adults were related to the mental health status of older adults, and the regression results revealed that sex, age, type of household, residence type, drink or not, whether to exercise, pension insurance, and annual household income were the most important factors influencing the mental health status of older adults, which was consistent with the results of existing studies (28). As the senior age, their physical functions gradually decline, thus cognitive functions are easily impaired, female seniors have deviations in mental health compared to male seniors, rural seniors have greater differences in living environment and living conditions compared to urban seniors, have single daily activities and less exposure to information (29), and are at greater risk of declining mental health levels, the lack of life care and emotional communication from spouses or children for older adults living alone or in institutions increases the risk of anxiety and depression. Participation in social activities and exercise can improve the physical health of older people, while maintaining social connections and avoiding social isolation, so that the risk of depression and anxiety decreases and social integration of older people is promoted.

The results of Bootstrap method proved that health care facilities has a partial mediating effect in the mental health status of older people with the ratio of mediating effect to total effect of 40.16%, indicating that the effect of spiritual comfort services on the mental health of older people may be effected through health care facilities.

Previous studies have shown that loss of health care facilities in older adults such as lack of social support, social isolation, and poor family relationships is associated with increased mortality, poorer self-rated health, lower quality of life, and higher risk of dementia (30, 31). It can also have profound negative effects on the health of older adults, such as psychological vulnerability (decreased cognitive, emotional, and coping abilities) and social vulnerability (decreased social relationships and social support), while older people with poor health and physical dysfunction are also more likely to feel lonely as well as more sensitive and dependent on the resources of their surroundings, thus affecting the wellbeing of older individuals. In older age groups, older adults with more social relationships and social involvement have better mental health status and physical health (32), which can improve their family relationships and social support to alleviate depressive symptoms in older patients. Consistent with the results of this study, by promoting the health care facilities of older adults, thus inhibiting or slowing down the generation of negative psychological emotions and improving their physical and mental health.

The use of spiritual comfort services can improve the mental health of older adults, effectively reducing and alleviating their mental health maladies, while better addressing the medical, psychological, cognitive and social needs of older adults and other patients with serious illnesses. Some studies have shown that spiritual comfort services not only improve the mental health of older people and effectively alleviate the feelings of loneliness and anxiety that occur in old age, but also improve the quality of life as well as the subjective perception level of older people (33). It is also believed that helping older people to eliminate the distress and anxiety in their hearts by communicating with them can alleviate their negative emotions such as loneliness and depression (34), which can play a positive spiritual and psychological role especially for the empty nesters or those whose children are unable to provide home care for older people due to their busy work schedules.

Moreover, the content of the spiritual comfort services should match the spiritual needs of older people, and when the community senior care needs are responded to, the utility of older people will be improved, thus satisfying their multi-level diversified needs in physical, psychological and cognitive aspects and improving the ability of older people to resist physical and mental health vulnerability (35). The community is an important guarantee for older people to enjoy socialized senior services, and the supply of community-based spiritual comfort services may be an important way to improve the mental health of older people, delay the trend of declining self-care ability of older people, and increase the effectiveness of socialized senior services (36). This finding suggests that community-based spiritual services are a key social factor influencing the mental health of older adults, as well as alleviating the stress of family caregiving and being an effective measure to address the health problems of older adults and improve their quality of life.

Based on the above research findings, the study makes some policy recommendations. We should carry out ”mental health screening“ for older people and rely on the community platform for accurate management. On the basis of the mental health assessment of older people, the service items, contents and standards are reasonably determined. To develop specific care and nursing intervention programs for the mentally unhealthy older people, including early determination of the degree of unhealthiness, timely assessment and active treatment, health education for older people, and design of more effective preventive measures for psychological intervention, social support and self-care ability. Orienting on the actual needs of older people, focusing on older people who are unable to take care of themselves, achieving a precise match between service supply and the needs of older people, focusing on disease prevention and management, active aging and social participation, etc., focusing on guiding older people to establish a positive view of aging, and making policy interventions in terms of improving the accessibility of medical care and the level of informal support for older people.

### Limitations

This study has the following limitations: first, there is a lack of period differences and age differences in the effects of mental comfort-type senior services on the mental health of older people; second, in reality, the mental health status of older people is the result of a combination of multiple influencing factors, and a more convincing indicator system is needed to measure the mental health of older people; third, because this study uses secondary data for analysis, it is limited by the variables of primary data, and only studies the differences at the individual level, and lacks the differential effects between different regions, different economic conditions and different living standards.

## Conclusion

In conclusion, this study uses cross-sectional data and structural equation modeling to examine the impact of spiritual comfort services in a community setting on the level of mental health of older adults and its impact on effectively reducing and mitigating their mental health vulnerability while better addressing the medical, psychological, cognitive, and social needs of older adults and other patients with serious illnesses.

Communities should optimize spiritual comfort services to maximize the effect of alleviating poor mental health and improving mental health by preventing or delaying the progress of poor mental health, so as to improve the physical and mental health of older people, improve the quality of life and mental health of older people, and promote healthy aging.

## Data availability statement

Publicly available datasets were analyzed in this study. This data can be found at: https://opendata.pku.edu.cn/dataset.xhtml?persistentId=doi:10.18170/DVN/WBO7LK.

## Ethics statement

The studies involving human participants were reviewed and approved by Medical Ethics Committee of Yunnan First People's Hospital. Written informed consent for participation was not required for this study in accordance with the national legislation and the institutional requirements.

## Author contributions

JD conducted field survey, collected data, provided suggestions, and manuscript preparation. YL conducted data collection, intervention, statistical analysis and manuscript design, writing, and editing. XZ conducted field survey, advised, conceived, and designed the work and edited the final version of the manuscript. ZW analyzed data and drew figures. YY provided suggestions, assisted in an English manuscript, and edited the manuscript. JD, YL, and XZ have full access to all the data in this study and takes primary responsibility for the final content. All authors have read and approved the final version of the manuscript.
